# Parent experiences of inpatient pediatric care in relation to health care delivery and sociodemographic characteristics: results of a Norwegian national survey

**DOI:** 10.1186/1472-6963-13-512

**Published:** 2013-12-10

**Authors:** Erling Solheim, Andrew M Garratt

**Affiliations:** 1 Norwegian Knowledge Centre for the Health Services, Postboks 7004 St Olavs plass, 0130 Oslo, Norway

**Keywords:** Parent experiences, Parent satisfaction, Quality of care, Inpatient pediatric care, Norway

## Abstract

**Background:**

The national survey of parent experiences with inpatient pediatric care contribute to the Norwegian system of health care quality indicators. This article reports on the statistical association between parent experiences of inpatient pediatric care and aspects of health care delivery, child health status and health outcome as assessed by the parents, and the parents’ sociodemographic characteristics.

**Methods:**

6,160 parents of children who were inpatients at one of Norway’s 20 pediatric departments in 2005 were contacted to take part in a survey that included the Parent Experience of Pediatric Care questionnaire. It includes 25 items that form six scales measuring parent experiences: doctor services, hospital facilities, information discharge, information about examinations and tests, nursing services and organization. The six scales were analyzed using OLS-regression.

**Results:**

3,308 (53.8%) responded. Mean scores ranged from 62.81 (organization) to 72.80 (hospital facilities) on a 0–100 scale where 100 is the best possible experience. Disappointment with staff, unexpected waiting, information regarding new medication, whether the staff were successful in easing the child’s pain, incorrect treatment and number of previous admissions had a statistically significant association with at least five of the PEPC scale scores. Disappointment with staff had the strongest association. Most sociodemographic characteristics had weak or no associations with parent experiences.

**Conclusions:**

The complete relief of the child’s pain, reducing unexpected waiting and disappointment with staff, and providing good information about new medication are aspects of health care that should be considered in initiatives designed to improve parent experiences. In the Norwegian context parent experiences vary little by parents’ sociodemographic characteristics.

## Background

Patient perceptions of quality of health care delivery that includes both experiences and satisfaction is an important component in health care evaluations, interventions and assessment of service quality [1]. Satisfaction with health services leads to better treatment adherence [2] which improves health outcomes [3]. The measurement of parents’ experiences and satisfaction with pediatric health care is a research area that has grown rapidly during the two last decades [2-21]. The two concepts of parent satisfaction and parent experiences are often used interchangeably in the literature, but in terms of operational definitions (variables) the former has often been assessed as a global measure, sometimes with just one item (question), whereas the latter asks more specifically about concrete experiences with the healthcare services. Such measurement usually takes the form of self-completed questionnaires administered to parents by means of a postal or clinic survey after the child has received an episode of care. The questionnaires often measure parent experiences using a number of scales relating to specific aspects of health care delivery, for example, doctor services, which could represent how parents perceive the doctor’s competence, interest in their child’s situation and communication abilities [4].

In spite of an increased availability of these questionnaires there has been little research assessing what variables are associated with parent experiences or satisfaction with pediatric care [4]. The comparison of parent experiences scores derived from a validated questionnaire with variables relating to health care structure and process will help inform the delivery of care so as to improve satisfaction levels. Previous research has largely focused on specific age groups or aspects of pediatric care, for instance neonatal intensive care [5,12], or has been limited to a single unit within a hospital [13] or one or a few hospitals [2,14,15]. These results may not be generalizable to other types of pediatric care or larger populations, including the national level. National representative surveys with large sample sizes are not common within the field of parent experiences [4], but such large-scale surveys of parent experiences are necessary when the aim is to inform quality improvement initiatives at the state or national level.

There have been two systematic reviews of the determinants of patient experience and satisfaction in general [1,16]. The first included 139 articles and was based on a structural framework which distinguished two broad groups of determinants: patient characteristics including beliefs, expectations, health status and values; and, health services delivery including human resources, organization of care and the physical care environment. Satisfaction was found to be significantly associated with communication and the relationship between patient and practitioner. Patient satisfaction also increased with utilization, how patients’ desires were granted, higher levels of health status and outcomes and age [1]. The second review considered associations between patient experience and clinical safety and effectiveness and included 55 studies [16]. Consistent statistically significant positive associations were found for these variables across a range of diseases, study designs and health care settings. Positive associations with patient experiences were found for objective and subjective measures of health outcomes, medication and treatment adherence, preventative care, resource use (hospitalization, length of stay and primary care visits), technical aspects of quality of care delivery and adverse events [16].

No such review has been undertaken for pediatric care and parent experiences or satisfaction. Moreover, there are few large-scale surveys involving multiple hospitals or clinics relating to parent satisfaction or experiences with pediatric care. Two nationally representative surveys within pediatric care from the United States have compared parent satisfaction or experiences with several variables by means of multivariate analysis [10,11]. The first study included three variables relating to parent experiences with well-child visits; a global satisfaction rating, satisfaction with information and satisfaction with time spent with the provider [10]. Spanish speaking mothers were found to have significantly lower odds than non-Hispanic white mothers of reporting information and time satisfaction. Parents who had missed or experienced delayed care had significantly lower odds of global and time satisfaction. Longer visit lengths were associated with higher levels of satisfaction for all measures [10]. The second study included parent ratings of global satisfaction and overall ease of using health care services as dependent variables in multivariate analyses. Compared with white parents, black parents were significantly more likely to report problems with ease of use. Dissatisfaction with care and problems with ease of use were significantly associated with the severity of the child’s condition, lack of insurance, parental interview in Spanish and inadequate family-centered care [11].

The lack of national representative surveys of parental experiences and heterogeneity in variables across studies makes it difficult to develop a theoretical framework and deduce hypotheses from the existing literature. This study includes six scales assessing different aspects of parental experiences with pediatric care that have structural validity; doctor services, information relating to discharge, hospital facilities, information relating to examinations and tests, nursing services and organization [22]. This allows for a more detailed evaluation of experiences than a single question relating to overall satisfaction or a global measure derived from the six scale scores. We also include other aspects of parent experiences with the health care delivery as independent variables in our study, such as experiences with waiting time. Parents have been asked to report on aspects of health care such as waiting times in previous studies of parent satisfaction with pediatric care [19]. However, this methodological approach limits the interpretation of results to reporting on statistical associations rather than causality. The analyses that follow test the same model for each scale. This study of parental experiences with inpatient pediatric care is the first national representative study of its kind in Norway. Hence, the a priori hypotheses are based on findings from the literature on patient experiences more generally and studies where the target population is the parents of children of a certain age or diagnosis, including populations that are not Norwegian and not nationally representative. Hence the approach taken is to some extent explorative.

The current study is representative of the Norwegian context. In Norway all pediatric departments are publicly administered at the regional level and all children have equal access to health care. These are universal policy rights guaranteed by the state, and parents do not need health insurance to gain access to pediatric care. Compared with most other developed countries the Norwegian population is relatively homogeneous in terms of income levels, for instance between unskilled workers and professionals, and the welfare state provides fairly generous benefits to those who cannot work. Since the early 1970s immigration has increased, but by 2005 Norway still had a small proportion of first or second generation immigrants.

## Methods

### Data collection

The Norwegian national survey of parent experiences of inpatient pediatric care is based on a validated questionnaire that measures parent experiences with inpatient pediatric health care, the Parent Experiences of Paediatric Care (PEPC) questionnaire [22], together with questions relating to parent perceptions of child health status, health outcome, some other aspects of the health care delivery, and sociodemographic variables. The survey was carried out by the Norwegian Knowledge Centre for the Health Services, an independent organization funded by the Norwegian Ministry of Health and Social Affairs. The questionnaire was mailed one or two weeks after discharge to 6,160 parents of children aged up to 16 years who had been inpatients at one of the 20 pediatric departments across Norway. This was based on drawing a probability sample of 400 patients selected from each department. Data were collected during a twelve week period from 15 September 2005. Units providing pediatric care for habilitation, rehabilitation, neonatal or psychiatric care and units for adults offering pediatric care were excluded. All questionnaires were written in Norwegian. One reminder was sent after four weeks [23].

The Norwegian Regional Committee for Medical Research Ethics, the Data Inspectorate and the Norwegian Board of Health approved the survey in accordance with the Helsinki Declaration of 1975, as revised in 1983.

### Study variables

#### Dependent variables

The PEPC questionnaire includes 25 items with five-point scales that sum to produce six scales of parent experiences with scores from 0 to 100 where 100 is the best possible experience. The items are scaled from ‘not at all’ to ‘to a very large extent’ with the exception of the hospital facilities scale where items have scaling from ‘very poor’ to ‘very good’. The PEPC questionnaire has good evidence for data quality, internal consistency, test-retest reliability and validity [22], and it is available from the Norwegian Knowledge Centre for the Health Services website [23].

The scale *doctor services* comprises five items relating to whether the parent felt the doctor showed care and interest in him or her and the child, an interest in listening to his or her opinions, if the explanations were easy to understand, and whether the doctor seemed competent (Cronbach’s alpha = .90). *Information relating to discharge* comprises three items relating to whether the respondent received sufficient information before discharge, if the journey home was safe and if there was sufficient information on how to proceed if anything was to happen after discharge (Cronbach’s alpha = .78). *Hospital facilities* comprises four items relating to the hospital’s physical environment and facilities including cleanliness, bathroom facilities, quietness, rest room and overnight facilities (Cronbach’s alpha = .73). *Information relating to examinations and tests* comprises two items about the parent’s understanding of how the tests and examinations were conducted and whether related information was sufficient (Cronbach’s alpha = .80). *Nursing services* comprises seven items about help with caring for the child, care and support for the parents, information about their role, interest in the parent’s opinions, family situation considerations, ease of understanding the information received, and care for the child (Cronbach’s alpha = .88). Finally, *organization* comprises four items including the extent of continuity of care by one doctor alone, continuity of health care personnel, whether personnel co-operated when treating and nursing the child, and if the treatment followed a thorough plan (Cronbach’s alpha = .78).

### Independent variables

In addition to the 25 items used to construct the six PEPC scales, the questionnaire also included items measuring parent experiences with aspects of health care delivery. Four items asked whether the parent had felt angry, disappointed or upset with the hospital staff (henceforth disappointed with staff), experience with waiting, perceived incorrect treatment and information relating to medication. These were all rated on a five-point scale from ‘not at all’ to ‘to a large extent’. If the child had not received any new medication the parent could answer ‘irrelevant’. The number of previous admissions in the last two years ranged from ‘none’ to ‘more than ten’. Whether staff eased pain was rated ‘yes, fully’, ‘yes, partly’, ‘no’ and ‘irrelevant’.

The parent’s perception of the child’s health status was rated on a five-point scale from ‘poor’ to ‘excellent’ and perception about the child’s health outcome after treatment on a five-point scale from ‘a lot worse’ to ‘much better’. Whether the parent who stayed at the hospital received help or not from friends or family with child care for any other child was assessed as ‘yes’, ‘no’, ‘required no help’ and ‘does not have any other children’. If the parent was alone with the child at the hospital was assessed as ‘yes’ or ‘no’.

The questionnaire included several sociodemographic questions for the parent (respondent) and any second adult in the household. The respondent was asked if he or she was the mother, the father, if the mother and father answered the questionnaire together, or if he or she was someone else than a parent (e.g. grandparent). Ethnicity included being of Norwegian or Sami, European or non-European origin. Education was assessed as highest level achieved from primary, secondary (high school), graduate (bachelor) and postgraduate (master/PhD). Main economic activity included ‘salary working’, ‘home working’, ‘education’, ‘disability pension’ and ‘other’ (e.g. unemployed). Marital status included ‘married’, ‘living with a partner’ and ‘single’.

#### Covariates

The age and gender of the child and the respondent’s age were included as covariates. Type of treatment included ‘medical’, ‘surgery’ or ‘other’.

The variables were either recoded into sets of binary dummies with ‘irrelevant’, ‘do not know’ or a substantial category as the reference category, or treated as continuous variables when applicable (see also Table 1).

**Table 1:** Descriptive statistics of variables with 95% CI for PEPC scales (N = 2872)

	**Means and standard deviation**	**95% CI**	**Min**	**Max**
Nursing services^i^	64.05 (19.57)	(63.33–64.76)	0	100
Doctor services^i^	69.96 (19.41)	(69.25–70.67)	0	100
Organization^i^	62.81 (19.68)	(62.08–63.53)	0	100
Information – discharge^i^	71.41 (20.49)	(70.66–72.16)	0	100
Information tests^i^	69.51 (21.10)	(68.74–70.28)	0	100
Hospital facilities^i^	72.80 (19.24)	(72.10–73.51)	0	100
Child’s age	5.22 (4.55)	(5.05–5.39)	0	16
Respondent’s age	34.89 (6.55)	(34.65–35.12)	16	67
Child’s health status now^vi^	3.67 (1.11)	(3.62–3.70)	1	5
Health worsened or improved^vii^	4.25 (.95)	(4.21–4.28)	1	5
Times hospital last two years^v^	1.78 (1.12)	(1.74–1.82)	1	5
Unexpected waiting^iv^	2.57 (1.14)	(2.53–2.62)	1	5
Incorrect treatment^iv^	1.27 (.68)	(1.24–1.29)	1	5
Disappointed at staff^iv^	1.84 (1.12)	(1.79–1.88)	1	5
	**Percentages**			
Gender (girls = 1)^ii^	43.18%			
Ethnicity^iii^				
Norwegian/Sami	Referent			
European	2.89%			
Non-European	2.79%			
Education^iii^				
Primary	Referent			
Secondary	41.05%			
Graduate	33.18%			
Post-grad.	19.08%			
Main activity^iii^				
Other/missing	Referent			
Disability pension	1.53%			
Education	5.29%			
Home working	17.69%			
Salary working	60.76%			
Marital status^iii^				
Married	Referent			
Partner	30.01%			
Single	11.56%			
Information on new medication^iii^				
Irrelevant	Referent			
Not at all	5.40%			
Small extent	10.13%			
Some extent	15.08%			
Large extent	15.98%			
Very large extent	6.55%			
Staff eased pain^iii^				
Irrelevant/don’t know	Referent			
Yes, fully	54.35%			
Yes, partly	17.79%			
No	3.86%			
Type of treatment^iii^				
Medical	Referent			
Chirurgical	19.22%			
Other	18.98%			
Help friends/family^iii^				
No other child	Referent			
Yes	12.05%			
No	21.55%			
Not alone with child^ii^	48.54%			

Notes: PEPC indicates parent experiences with pediatric care.; CI, confidence interval.

^i^PEPC scales are scored from 0 to 100 where 100 is the best possible parent experience. ^ii^Binary dummies. ^iii^Binary dummies recoded for each category. ^iv^Likert type scales 1–5, from ‘not at all’ to ‘to a large extent’. ^v^Scale: one, two, three to five, six to ten, more than ten. ^vi^Scale 1–5, from ‘poor’ to ‘excellent’. ^vii^Scale 1–5, from ‘much worse’ to ‘much better’.

### Hypotheses

The hypotheses based on expected associations between the six PEPC scale scores and the independent variables, relate to the two broad groups of determinants of experiences or satisfaction with care; health services delivery and patient characteristics [1]. Regarding the former, information provision has been found to have an association with care experiences and it is hypothesized that information about new medication will have a positive association [1,17,18]. There is consistent evidence that the relationship with health personnel is important for patient satisfaction [1] and it was hypothesized that disappointment with staff would be associated with poorer experiences. Patient and parent satisfaction has been found to be adversely affected by waiting times [1,19] and it was hypothesized that unexpected waiting would be associated with poorer experiences. Pain management has been found to contribute to parent satisfaction [20] and it was hypothesized that the easing of the child’s pain would be associated with better experiences. It was hypothesized that incorrect treatment would be associated with poorer experiences [1]. Finally, it was hypothesized that the number of hospitalizations in the last two years would be positively associated with parent experiences [11].

With regard to parent and patient characteristics and following previous findings, it was hypothesized that more positive parent experiences will be positively associated with an older respondent age and better levels of parent-reported child health and outcomes [1,10,11]. Social support can be important for families receiving health care and may influence the parents’ experiences. It was hypothesized that parents with a partner or married, those receiving help from friends or family with a second child (if any), and those who were not alone at the hospital would have more positive experiences compared to those with less social support.

There were no specific expectations about the direction of association between parent experiences and the child’s age or gender, the respondent’s gender and type of treatment, but these variables were chosen in the model as potential determinants of parent experiences. No such associations would mean that the results would not be limited to or more relevant for children of specific ages or who receive a specific type of treatment, or dependent on which parent answered the survey.

Weak associations are hypothesized between parent experiences and sociodemographic characteristics (education, ethnicity, economic activity) because of the homogeneity of the Norwegian population and universal equal access to health care. Sociodemographic characteristics of the second adult (if any) in the household are hypothesized to have the same associations as the sociodemographic characteristics of the respondent. However, as the parents are likely to have similar sociodemographic characteristics, it is possible that the variables relating to the second adult in the household (if any) are non-significant after controlling for the respondent’s sociodemographic characteristics.

### Statistical analysis

The six PEPC scale scores were regressed on the same model of independent variables and covariates using multiple OLS-regression. One regression model was estimated for each of the six PEPC scales. Coefficients are reported as standardized regression coefficients, ranging from -1 to +1. T-tests were used to test the association of single coefficients and nested models F-tests to test the association of variables recoded into a set of dummies. All tests were two-tailed. The sample size was kept constant within each regression as each nested model was tested while controlling for all other independent variables and covariates in the model. The independent variables that were statistically non-significant in all six regressions were dropped from the model. The results presented are based on the final model after dropping the non-significant variables.

## Results

### Data collection

Of the 6,160 parents sent a questionnaire, 3,308 (53.8%) responded; 71.4%, 11.9%, 16.1% and 0.6% were the mother, father, both parents or another person respectively. Of these, 93.6% were ethnic Norwegians, 3% had European and 3.4%, a non-European ethnic origin. The response rate varied from 49% to 61% among the departments. Differences between the gross and net samples were assessed on the basis of patient information and not the characteristics relating to the parents, because the hospital records do not include the latter. The only statistically significant difference between respondents and non-respondents was related to the patient’s length of stay; 2.95 days compared to 2.60 days for those who did not respond. There was no difference based on the child’s age or gender, but when compared with the educational level among Norwegian adults in general, it is possible that parents with higher education are slightly over-represented in the sample [23]. There were no significant differences across the six PEPC scale scores based on the child’s age, gender or respondents who received a reminder or not [23]. Due to missing data, the sample used in all analyses comprises 2872 cases.

### Descriptive analysis

Table 1 shows descriptive statistics with a 95% confidence interval of the mean for the dependent and independent variables. Mean PEPC scores ranged from 62.81 (organization) to 72.80 (hospital facilities) and the standard deviations were large across the scales which suggests a large potential for improving how Norwegian parents experience the health care delivery process in inpatient pediatric care. The children’s mean age was 5.21 years and 43.4% were female. The majority of children received medical treatment and just over 40% had been in hospital more than once over the past two years. 58% of parents perceived their child’s health to be very good or excellent, 25% good and 17% bad or very bad. Most parents thought their child’s health improved some or much due to the treatment received. However, nearly 17% of parents thought that their child had received incorrect treatment, and about 44% felt disappointed with the staff in some way.

Table 2 presents Pearson’s r correlations for the six PEPC scales. Hospital facilities have the lowest correlations with the other five scales, ranging from .27 to .44. All other correlations ranged from .48 to .62, suggesting a moderately strong association between the scales. The strength of these correlations suggests the PEPC scales are associated but indeed measure different aspects of parent experiences. This results support the approach of constructing multiple scales measuring different aspects of parent experiences, and the statistical associations between each respective PEPC scale and the independent variables in the model is likely to vary across the six regressions.

**Table 2:** Pearson’s r correlations between PEPC scales (N= 2872)

	**Doctor services**	**Hospital facilities**	**Information discharge**	**Information tests**	**Nursing services**
Hospital facilities	.28***				
Information discharge	.56***	.27***			
Information tests	.62***	.29***	.53***		
Nursing services	.62***	.44***	.49***	.60***	
Organization	.59***	.34***	.48***	.58***	.61***

Note: ***p < .001.

### Multivariate analyses

The variable measuring the type of respondent (mother, father, other) and all sociodemographic variables relating to the second adult (if any) were non-significant in all six regressions and were therefore excluded from the final model regressed on the six PEPC scales. The regression results are shown in Table 3. The independent variables with the strongest associations were disappointment with staff, information relating to new medication, staff eased pain and unexpected waiting. These associations were statistically significant in all six regressions. Disappointment with staff had the strongest association in all six regressions. Increasing disappointment with staff and unexpected waiting was associated with less positive experiences on all six PEPC scales.

**Table 3:** OLS regressions with PEPC scales as dependent variables (N = 2872)

		**PEPC Scales**				
**Characteristics**	**Doctor services**	**Hospital facilities**	**Information discharge**	**Information tests**	**Nursing services**	**Organization**
Child’s age	-.03	.09***	-.00	.05*	.03	.03
Gender (girls = 1)	-.02	-.04*	.01	.00	.01	.00
Age respondent	.06**	-.03	.04	.03	.01	.03
Ethnicity	**1.45**	**2.72**	**.14**	**.42**	3.08*	6.67**
Norwegian/Sami	Referent	Referent	Referent	Referent	Referent	Referent
European	.01	.01	.00	.01	.01	.02
Non-European	.03	.04*	.01	.01	.04*	.06***
Education	**.31**	**1.16**	**.13**	**.32**	**.91**	3.10*
Primary	Referent	Referent	Referent	Referent	Referent	Referent
Secondary	-.03	-.03	-.02	-.03	-.01	-.01
Graduate	-.02	-.05	-.01	-.03	-.01	-.04
Post-grad.	-.03	-.02	-.01	-.02	.02	-.06*
Main activity	**1.95**	**1.24**	3.93**	4.38**	5.64***	2.47*
Other/missing	Referent	Referent	Referent	Referent	Referent	Referent
Disability pension	-.01	-.01	-.03	-.00	-.01	-.02
Education	-.00	-.03	-.05*	-.02	-.02	.00
Home working	-.04	.01	-.05*	.01	-.03	.01
Salary working	-.06*	-.03	-.09***	-.07**	-.10***	-.05*
Marital status	**.69**	**.17**	**.15**	**.33**	**.44**	4.17*
Married	Referent	Referent	Referent	Referent	Referent	Referent
Partner	-.02	.00	-.00	-.01	-.00	.02
Single	.00	.01	.01	.00	.01	.05**
Info on new medication	47.96***	8.39***	41.09***	64.68***	46.40***	27.56***
Irrelevant	Referent	Referent	Referent	Referent	Referent	Referent
Not at all	-.10***	-.03	-.09***	-.14***	-.12***	-.07***
Small extent	-.11***	-.09***	-.10***	-.13***	-.09***	-.06***
Some extent	-.06**	-.06**	-.06**	-.05**	-.04*	-.03
Large extent	.05**	.00	.07***	.06**	.04*	.04*
Very large extent	.18***	.05*	.16***	.19***	.17***	.14***
Staff eased pain	13.40***	4.60**	8.25***	22.69***	34.78***	20.39***
Irrelevant/don’t know	Referent	Referent	Referent	Referent	Referent	Referent
Yes, fully	.09***	.04	.07**	.14***	.14***	.11***
Yes, partly	-.03	-.03	-.03	.02	-.02	-.01
No	-.01	-.03	-.01	-.04*	-.07***	-.05**
Type of treatment	**.66**	14.24***	**1.59**	**1.98**	**.75**	**.06**
Medical	Referent	Referent	Referent	Referent	Referent	Referent
Surgery	.01	-.10***	-.02	.03	-.02	.00
Other	.02	-.01	-.03	-.00	.01	-.00
Help friends/family	**.97**	**2.04**	4.81**	**1.60**	2.79*	**1.69**
No other child	Referent	Referent	Referent	Referent	Referent	Referent
Yes	-.02	.04	-.00	.03	.03	.05*
No	-.03	-.00	-.05**	-.00	-.02	.02
Wished no help	-.03	.04	.02	.04	.02	.04
Not alone with child	.03	.02	.03	.05**	.06***	.03*
Child’s health status	.03	.05*	.17***	.05**	.03	.05*
Health worsened/impr.	.02	.02	.06**	.02	.02	-.01
Times hospital last 2y	.05**	-.12***	.10***	.06***	.01	.08***
Unexpected waiting	-.11***	-.11***	-.07***	-.15***	-.16***	-.20***
Incorrect treatment	-.11***	.04*	-.09***	-.09***	-.00	-.09***
Disappointed with staff	-.25***	-.20***	-.19***	-.21***	-.28***	-.29***
F	35.99***	17.00***	31.40***	43.15***	44.92***	47.51***
Adjusted R-square	.29	.16	.27	.33	.34	.36

Notes: PEPC indicates parent experiences with pediatric care.; F-statistic and statistical significance for each of the nested model F-tests in bold. Beta coefficients. Statistical significance: *p < .05; **p < .01; ***p < .001.

The two variables ‘information relating to any new medication’ and ‘whether or not the staff managed to ease the child’s pain’, showed two distinctive patterns of associations across the regressions. Both variables were recoded into sets of dummy variables with those who responded that these two questions were irrelevant to them, i.e. their child did not receive any new medicine or did not feel pain, forming the reference category. The two distinctive patterns appear as a change in the direction of association between parents who felt they received some rather than a large amount of information and those parents who felt the staff eased their child’s pain fully rather than only partly or not at all. These results suggest a threshold level of the quality of health care delivery necessary for parents requiring information for new medicines or whose child suffers from pain to have a positive experience as assessed by the six PEPC scales. Compared with parents whose children did not receive new medicine, parents whose children did needed to perceive they received a large extent of information or more to have a positive experience of care. Compared to parents whose children did not have pain, parents with children who suffered from pain need to perceive on average that health personnel staff eased their child’s pain fully in order to have a positive experience of care across the six PEPC scales.

Incorrect treatment and number of times the child had been in hospital during the two last years had statistically significant associations with all PEPC scales except nursing services. With a few exceptions, the associations for these variables were not as strong as those above. In the four regressions when doctor and nursing services were not the dependent variables, poorer child health status was associated with more negative experiences. Number of times in hospital was associated with more positive parent experiences in four regressions, but was negatively associated with hospital facilities. Incorrect treatment had a negative association in four regressions, and was positively associated with hospital facilities. Incorrect treatment had a negative association in three regressions, and was positively associated with hospital facilities. This means that both numbers of times in hospital and incorrect treatment had an association with hospital facilities that went in the opposite direction of what was hypothesized. Not alone with child at the hospital had a statistically significant association with three PEPC scales.

Help from friends or family and type of treatment had a weak association with two and one PEPC scales respectively. The former showed no discernible pattern between those receiving help, those who did not receive help and those who did not want any help. Parents of children receiving medical treatment had slightly better experiences than parents of children receiving surgical treatment for the PEPC scale of hospital facilities.

For the sociodemographic characteristics only the parent’s main activity showed a pattern across regressions. Parents whose main activity was salary work reported more negative experiences than other parents in four of the regressions, the exceptions being when doctor services and hospital facilities were the dependent variables. Ethnicity had two significant associations. Ethnic non-Europeans had more positive experiences than ethnic Norwegians regarding nursing services and organization. Marital status and educational level had one significant association. The highly educated and single parents had more negative experiences with organization.

Only the regression with organization as the dependent variable had four significant associations relating to the sociodemographic characteristics. This regression also had the highest Adjusted R-square (explained variance) of the six regressions and hence the model best explains most variation in parent experiences with organization of the health care. The regression with hospital facilities as the dependent variable had the lowest Adjusted R-square. This PEPC scale also had the lowest correlations with the other five PEPC scales, suggesting hospital facilities is an aspect of parent experiences standing out from the five other aspects and that the model is poor at predicting variation in parent experiences with hospital facilities.

## Discussion

This large national representative study is the first to assess parent experiences of inpatient pediatric care in Norway in relation to aspects of health services delivery, the child’s health status and health outcome as assessed by the parent and sociodemographic variables by means of multiple regression analysis. The study used a validated questionnaire as part of a national survey of parents whose children attended the 20 Norwegian hospitals with a pediatric department. The response rate of 53.8% is acceptable in comparison to previous studies of parent experiences or satisfaction that used similar survey methods [22], and based on comparisons of patient characteristics between the gross- and net samples, there is little reason to believe the sample is not representative for the population. However, the response rate is lower than in most studies where parents have been approached in a more direct way, e.g. at the clinic [22]. Questionnaires and reminders were mailed from the Norwegian Knowledge Centre for the Health Services, an independent organisation funded by the Ministry of Health and Social Affairs. ‘Personal contact’ or more direct approaches which include surveying parents as part of initial and follow-up procedures at the hospital department might have produced higher response rates, but with a larger potential for social desirability bias [24,25]. Parents may report better experiences because they feel that this will be more acceptable to the hospital department responsible [21]. The questionnaire was only available in Norwegian language, and as a consequence non-Norwegian speakers may not have answered the questionnaire. The average of highest achieved level of education was somewhat higher in the data compared to that for the Norwegian population in general [23]. It is therefore possible the survey was less representative for immigrants who do not read Norwegian well and those with low education.

The means for the PEPC scales ranged from 62.81 to 72.80, with standard deviations from 19.24 to 21.10. This suggests most parents had good experiences with Norwegian inpatient pediatric health care, but the large variation in experiences shows a large potential for improvements in several areas of health care delivery. These results are, with one exception [15], similar to other studies that report parents have mostly positive experiences with pediatric care [4].

Most of the standardized regression coefficients were either weak or very weak, and the large sample size increased the risk of a Type I error when testing each coefficient’s statistical significance. Hence caution is needed in interpreting the results which includes focusing primarily on patterns of statistically significant coefficients across the six models. The results for the PEPC scales of organization and hospital facilities are also interesting because they differ from those for the other PEPC scales. Organization had the greatest number of significant associations, including several associations with sociodemographic characteristics. Hospital facilities had the least explained variance and some associations were in the opposite direction to that hypothesized and observed for the other scales.

The six regressions showed that most of the variation and potential for improvement is associated with independent variables related to health care delivery rather than the child’s health, health outcome, social support and sociodemographic characteristics. The variables measuring parents’ disappointment with staff, unexpected waiting, information relating to new medication and whether staff were successful in easing the child’s pain were the strongest and statistically significant across all six regressions. These results are consistent with earlier studies in patient satisfaction more generally [1], showing the results relating to adult patients are relevant for understanding parent experiences with inpatient pediatric health care delivery.

Incorrect treatment and the number of times the child had been admitted to hospital in the last two years had statistically significant associations with all PEPC scales except nursing services. Similar questions have been used in parent and patient satisfaction studies more generally, and show moderate levels of association with satisfaction ratings, similar to the results reported here [1]. Incorrect treatment and number of admissions in the last two years share one inconsistency in the direction of associations. While the former had a negative association with all scales but hospital facilities, this pattern is reversed for the latter. Parents whose child had received incorrect treatment thus experience the hospital facilities more positively, while parents whose child has been hospitalized many times had a negative experience with the hospital facilities. It is difficult to interpret why parents whose child had received incorrect treatment were positive towards the hospital’s facilities. It is possible that parents with chronically ill children visit the hospital more often and hence may have a better understanding of the facilities, but the negative association between hospital facilities and number of hospital visits in the last two years contradicts such an interpretation. Also parents whose children had surgical treatment had more negative experiences with hospital facilities but not with any of the of the other PEPC scales. This suggests these parents are less satisfied with the physical environment, which is of potentially greater importance for these parents.

The more general patient satisfaction literature and a few parent satisfaction studies document a consistent positive association between good health and positive satisfaction [1,10,26-28]. The current study shows the same positive association exists between the child’s health as perceived by the parent and four of the PEPC scales, excluding doctor and nursing services. This suggests that child health status should be controlled for in future comparisons of health care providers as recommended in the satisfaction literature more generally [1,26-28].

Child’s health and health outcome were based on the parent’s evaluation. Self- or health professional ratings may be more valid and reliable. It is also possible that parents who had more negative experiences of health care might give more negative ratings of child health and outcomes. This would have improved the strength of association between parental experiences and health status or health outcomes, but it was found that only the association between child health status and information at discharge had a relatively strong association. Three of the other four significant coefficients were much smaller, and two coefficients were statistically non-significant. Health outcome was statistically significant in only one regression, information at discharge.

Another limitation with this study relating to health and parent experiences is the lack of control for parental health and parents’ experiences with health care delivery in relation to their own health. Future research should consider controlling for these variables, because the quantity and quality of experiences among parents as patients themselves could affect their expectations and experiences as parents when their children receive pediatric care [4].

Whether or not the parent is alone with the child at the hospital, has a partner or received help to care for other children (if any), had little impact on parent experiences. This can be interpreted in a positive way, because it shows that the parental experiences with inpatient pediatric care are in general little affected by the parent’s social support. The exemption is parents who were not alone with the child in the hospital. They had somewhat more positive experiences with information relating to examinations and tests, nursing services and organization.

The associations between the sociodemographic covariates and PEPC scale scores indicate that there is very little variation in parent experiences due to sociodemographic heterogeneity, with the exception of salary working parents who were more negative, and ethnicity, where non-European immigrants had more positive experiences with nursing services and organization. This latter result is interesting because it contrasts with previous research in the USA and Switzerland regarding patient satisfaction more generally [1] and in the USA regarding parent satisfaction [11]. It is possible that most parents with a non-European language as their mother tongue are first generation immigrants and therefore experience Norwegian pediatric health care more positively than in their country of origin. Alternatively, because the questionnaire was distributed in Norwegian only, immigrants who are the least integrated and do not speak the Norwegian language could be underrepresented in the survey, with the consequence immigrants with the least positive experiences did not participate.

The sociodemographic variable of education was only associated with parent experiences relating to organization, while economic activity had a weak association with four aspects of parental experiences. This result supports the hypothesis of weak associations between sociodemographic characteristics of socioeconomic status and parent experiences. This result should be interpreted in relation to the study population, which is representative for all patients who visited any of the Norwegian hospitals offering inpatient pediatric care, and how access to health care is covered by universal rights guaranteed by the state. The results show that despite including the entire population there was little variation in parent experiences, and perhaps somewhat surprisingly, it was those who are employed and not the more marginalized parents (e.g. those disabled with poor health and lower incomes) who report parent experiences more negatively. Future research should assess to what extent these results are consistent across countries that differ in terms of access to health care and the population’s composition in terms of ethnicity and income levels.

The regressions with the PEPC scales of doctor and nursing services as dependent variables had some associations that were fairly similar, but they differ regarding help from friends and family, alone with child at hospital, number of times at hospital the two last years, incorrect treatment and the three sociodemographic variables age, ethnicity and main economic activity. These results support the decision to measure doctor and nursing services separately, and health care delivery initiatives that aim to improve parent experiences and satisfaction in relation to staff services should take account of how parents distinguish between services provided by doctors and nurses.

All six models had a low amount of explained variance, with adjusted R-square measures ranging from .16 to .36. It is recommended that future research should extend the regression models with more theoretically relevant variables, including variables measuring hospital specific information. The number of hospitals was too small to include several variables measuring hospital specific information within a multilevel analysis framework, but this approach should nevertheless be considered in future research.

Moreover, a cross-sectional survey has its limits with consequences for theory and the construction of hypotheses. The respondents were used as informants to evaluate the child’s health status and other aspects of the health care delivery, such as waiting time. An alternative method would have been to use health professionals to evaluate the child’s health status and health outcome, and waiting time could have been monitored by other means than the parents’ self-reporting. Hence the study was limited to reporting statistical associations between the six aspects of parental experiences and the independent variables rather than testing causality.

## Conclusion

The Norwegian national survey on parent experiences of inpatient pediatric care found that parents’ experiences relating to disappointment with staff, unexpected waiting, information regarding new medication and staff’s ability to ease the child’s pain fully or not had a statistically significant association across six important aspects of parent experience as assessed by a validated questionnaire. Disappointment with staff had the strongest association across all regressions. Several other health care related variables and the child’s health status as perceived by the parents had an association with four or five PEPC scales. The results suggest these variables should be considered in the context of initiatives that are designed to improve parent experiences and satisfaction with pediatric care. In the Norwegian context of universal coverage and access to pediatric health care, and a population with relatively small income differences and few immigrants, sociodemographic characteristics of the parents had little impact on parent experiences.

## Abbreviations

PEPC: Parent experience of pediatric care questionnaire.

## Competing interests

The authors declare that they have no competing interests.

## Authors’ contributions

ES was responsible for the data analysis and writing the manuscript. AG was responsible for the design and execution of the study and contributed to the writing of the manuscript. Both authors read and approved the final manuscript.

## Pre-publication history

The pre-publication history for this paper can be accessed here:

http://www.biomedcentral.com/1472-6963/13/512/prepub
